# Xanthine Oxidoreductase Is Involved in Chondrocyte Mineralization and Expressed in Osteoarthritic Damaged Cartilage

**DOI:** 10.3389/fcell.2021.612440

**Published:** 2021-02-09

**Authors:** Sonia Nasi, Mariela Castelblanco, Véronique Chobaz, Driss Ehirchiou, Alexander So, Ilaria Bernabei, Teruo Kusano, Takeshi Nishino, Ken Okamoto, Nathalie Busso

**Affiliations:** ^1^Service of Rheumatology, Department of Musculoskeletal Medicine, Centre Hospitalier Universitaire Vaudois and University of Lausanne, Lausanne, Switzerland; ^2^Department of Biochemistry and Molecular Biology, Nippon Medical School, Tokyo, Japan

**Keywords:** calcium-containing crystals, osteoarthritis, animal model, chondrocyte calcification, xanthine oxidoreductase

## Abstract

Pathologic calcification of cartilage consists of the formation of basic calcium phosphate (BCP) and/or calcium pyrophosphate dihydrate (CPPD) containing calcium crystals in mature hyaline or articular cartilage and is associated with aging, cartilage injury and likely plays a role in accelerating the pathology of osteoarthritis (OA). The pathways regulating joint calcification, in particular cartilage calcification, are not completely understood, but inflammation and the formation of reactive oxygen species (ROS) are contributory factors. The xanthine oxidase (XO) form of xanthine oxidoreductase (XOR), the key enzyme in xanthine and uric acid metabolism, is a major cellular source of superoxide. We hypothesized that XOR could be implicated in chondrocyte mineralization and cartilage calcification and degradation in OA. We showed both in murine primary chondrocyte and chondrogenic ATDC5 cells, that mineralization was inhibited by two different XOR inhibitors, febuxostat and allopurinol. In addition, XOR inhibition reduced the expression of the pro-mineralizing cytokine interleukin-6 (IL-6). We next generated XOR knock-out chondrocyte cell lines with undetectable XOR expression and XO activity. XOR knock-out chondrocyte cells showed decreased mineralization and reduced alkaline phosphatase (Alp) activity. To assess the precise form of XOR involved, primary chondrocytes of XOR mutant mice expressing either the XDH form (XDH ki) or the XO form (XO ki) were studied. We found that XO ki chondrocytes exhibited increased mineralization compared to XDH ki chondrocytes, and this was associated with enhanced Alp activity, ROS generation and IL-6 secretion. Finally, we found increased XOR expression in damaged vs. undamaged cartilage obtained from OA patients and XOR expression partially co-localized with areas showing pathologic calcification. Altogether, our results suggest that XOR, via its XO form, contribute to chondrocyte mineralization and pathological calcification in OA cartilage.

## Introduction

The formation of calcium crystals [both basic calcium phosphate (BCP) and calcium pyrophosphate dihydrate (CPPD)] within adult human cartilage is pathological and is a contributory factor in the development and progression of osteoarthritis (OA) (Fuerst et al., 2009; McCarthy and Dunne, 2018; Yan et al., 2020). Calcification can be induced by cartilage injury, metabolic disturbances, aging and inflammation, but the precise mechanisms that regulate this process in chondrocytes is poorly understood. We have previously shown that the inflammatory cytokine IL6 (Nasi et al., 2016b), reactive oxygen species (ROS; Nasi et al., 2016a, 2020) as well as the cellular levels of the gasotransmitter hydrogen sulfide influence the calcification potential of chondrocytes *in vitro* and *in vivo*, and the severity of experimental OA (Castelblanco et al., 2020; Nasi et al., 2020). Formation of ROS within chondrocytes can play a role in ageing and OA via induction of metalloproteases (MMPs), inflammation and apoptosis (Lepetsos and Papavassiliou, 2016). We have previously shown that xanthine oxidoreductase (XOR) is a cellular source of ROS that impacts on inflammasome activation (Ives et al., 2015) as well as immune regulation (Kusano et al., 2019).

Xanthine oxidoreductase, a molybdopterin-containing enzyme, is transcribed from the *xdh* gene and exists in two interconvertible forms, xanthine oxidase (XO) and xanthine dehydrogenase (XDH). Both forms oxidize hypoxanthine/xanthine to uric acid. Whereas XDH uses NAD^+^ as an electron acceptor and produces NADH, XO uses as an electron acceptor molecular oxygen, mainly producing ROS such as superoxide (O_2_^–^) and hydrogen peroxide (H_2_O_2_). XOR is originally expressed as the XDH form, but is also found in biological fluids in the XO form (see for review Battelli et al., 2016). Moreover, the conversion of XDH to XO is possible via either irreversible proteolysis or by reversible oxidation of thiol groups at positions 535 and 992 (Nishino and Okamoto, 2015). Both ROS and uric acid generation are inhibited by pharmacological xanthine oxidase inhibitors such as allopurinol (used in the treatment of gout) (Day et al., 2016). The biology of XO can also be explored by genetic modifications of XOR. Recently, we generated mice expressing either the XDH form (XDH ki mice, generated by introducing W338A/F339L mutations in *Xdh* gene) or exclusively the XO form (XO ki mice, with a C995R mutation) and showed that the XO ki form generated greater levels of O_2_^–^ than wild type or XDH ki mice (Kusano et al., 2019). We have therefore investigated the role of XOR, a potential source of ROS, in chondrocyte calcification by pharmacological and genetic approaches and studied its link to calcification in OA cartilage samples obtained from patients undergoing knee replacement surgery.

## Materials and Methods

### Mice and Experimental OA

For chondrocyte isolation, C57BL/6 (Charles River) or XOR mutant knock-in (ki) mice, either XDH ki or XO ki, generated by introducing C995R mutation or W338A/F339L mutations in *Xdh* gene respectively were used (Kusano et al., 2019). For experimental OA, 12-weeks old C57BL/6 female mice were subjected to medial meniscectomy (MNX) of the right knee, while the contralateral knee was sham-operated as control (Kamekura et al., 2005). Two months after, mice were sacrificed, and knees fixed in 10% formol.

### Mouse Knee Histology

Knees were dissected and decalcified in EDTA for 20 days and embedded in paraffin. Sagittal sections (5 μm thick, 3 sections/mouse, spaced 70 μM apart) of the medial compartment were stained with Safranin-O and counterstained with fast green/iron hematoxylin.

### Human Cartilage

Cartilage from 4 OA patients (mean age 72 ± 10 years) undergoing knee replacement (K/L score = 4) was obtained from the Orthopedic Department (CHUV, Lausanne-CH). Macroscopically intact (undamaged) and damaged cartilage was dissected and fixed in 10% formol for immunohistochemical analysis.

### Immunohistochemical Analysis

The anti-collagen II (Ab79127) and anti-collagen X (GTX37732 from Genetex) were used after treatment of fixed cells (15 min in 4% paraformaldehyde) with hyaluronidase 5 mg/ml for 30 min at 37°C. MMP13 was detected with anti-MMP13 (Ab39012 from Abcam). Quantification of immunohistochemical staining was done by image binarization and normalization of positive pixels over total cells. At least three independent fields were quantified per condition. Murine and human XOR expression was evaluated using an anti-XOR rabbit polyclonal antibody (5 μg/ml final concentration, sc-20991, Santa Cruz Biotechnology Inc., Dallas, TX, United States) on knee cartilage paraffin section. Semi-quantitative scoring was performed by two independent observers based on the following scale in three different fields per sample: 0 (no XOR expression); 1(up to 20% positivity); 2 (between 20%-40% positivity); 3 (between 40%-60% positivity); and 4 (above 60% positivity).

### Murine Chondrocytes Isolation

Immature chondrocytes were isolated from 5 to 7 days old mice as described (Nasi et al., 2016b) and amplified for 7 days in DMEM + 10%FBS to reach chondrocyte differentiation (Gosset et al., 2008).

### ATDC5 Chondrogenic Cell Line

ATDC5 (Sigma), a murine chondrogenic cell line mimicking primary chondrocyte phenotype was cultured in DMEM/F12 + 5%FBS + 1%ITS (insulin-transferrin-selenium, Sigma). In particular these cells were able to mineralize (Newton et al., 2012).

### CRISPR/Cas9 XOR Knock-Out Generation of ATDC5 Clones

Design of single-guide RNA (sgRNA) was performed using the online tool http://tools.genome-engineering.org. We adopted two strategies targeting either a sequence which included the starting codon or a sequence in the active site to design the sgRNA for mouse Xdh (“starting” oligos 5′-CACCGCTCCGGCCGTCACGATGACG-3′, 3′-CGAGGCCGGCAGTGCTACTGCCAAAM-5′, “Active site” oligos: 3′-CACCGGGATCTTCTCCGGAGTGGCG-5′, 5′-CCCTAGAAGAGGCCTCACCGCCAAA-3′). As control, we used a sequence targeting luciferase as non-relevant sgRNA as described previously (Di Micco et al., 2016). The complementary oligopairs generated a *Bsm*BI compatible overhang. After annealing, they were ligated into *Bsm*BI restricted lentiCRISPRv2 vector at a 1:1 molar ratio. After confirmation of single copy inserts by sequencing, lentiviral particles were generated using second generation lentiviral packaging plasmids (pVSVg and psPax2) together with the above described XOR or control lentiCRISPRv2 plasmids in HEK cells. ATDC5 chondrocytic cells were infected at a MOI of 1 and subcloned after puromycin selection, by plating single cells by FACS sorting. Clones were screened for absence of XO activity and clones having loss of XOR expression by Western-blot were further selected, expanded and used in the experiments.

### Cell Proliferation

10^6^ cells were labeled with 5 μM solution of Cell Proliferation Dye eFluor^®^ 670 in PBS. FACS analysis was performed after 72 h.

### Crystal Detection in Articular Chondrocyte Cultures

Chondrocytes were cultivated for 24 h in DMEM with 10%FBS, supplemented with Secondary calciprotein particles (CPP) (50 μg/ml calcium) to induce calcification (Aghagolzadeh et al., 2017; Nasi et al., 2020). Cells were fixed in 10% formol and calcium-containing crystals stained with Alizarin red (Gregory et al., 2004). Calcium content was quantified by the QuantiChrom^TM^ Calcium Kit (BioAssay Systems) by reading absorbance at 612 nm using the Spectramax M5e reader (Molecular Devices).

### Hydroxyapatite Crystals and Secondary Calciprotein Particles

Hydroxyapatite (HA) crystals were synthesized, characterized (Prudhommeaux et al., 1996), and sonicated for 5 min in sterile PBS prior to experiment. CPP were synthesized as previously described (Aghagolzadeh et al., 2017). Briefly, calcification medium was generated by adding 10% FBS, 3.5 mM inorganic phosphate (2.14 mM Na_2_HPO_4_, 1.36 mM NaH_2_PO_4,_ Sigma), 1 mM calcium (CaCl_2_, Sigma), 1% penicillin/streptomycin and 1% l-Glutamine to DMEM. This medium was stored at 37°C for 7 days to generate secondary CPP. The medium was centrifuged at 25,000 × *g* for 2 h at 4°C. The resulting pellet was resuspended by vigorous shaking, and calcium content was measured as described above (QuantiChrom^TM^ Calcium assay kit).

### Alkaline Phosphatase Activity

Chondrocytes were cultured for 6 h, supernatant was removed and alkaline phosphatase (Alpl) activity was measured in cell lysate using a p-Nitrophenyl Phosphate assay (Alpl Assay Kit, Abcam, ab83369) and by reading absorbance at 405 nm.

### Real Time PCR Analysis

RNA was extracted (RNA Clean and Concentrator5, Zymoresearch), reverse transcribed (Superscript II, Invitrogen), and quantitative Real Time-PCR (qRT-PCR) with gene specific primers (Table 1) using the LightCycler480^®^system (Roche Applied Science) was performed. Data was normalized against *Gapdh* reference gene, with fold induction of transcripts calculated against control cells.

**TABLE 1:** Murine and human gene specific primers for qRT-PCR.

Gene	Forward primer (5′→ 3′)	Reverse primer (5′→ 3′)
*mXOR*	TGT CAA CCT CTT CGT GTC CC	GAC AAA ACA GAG CGT CAG CG
*mGapdh*	CTC ATG ACC ACA GTC CAT GC	CAC ATT GGG GGT AGG AAC AC
*hXOR*	GCC TAA CAG GAG ATC ATA AGA ACC T	CTG GAC AAA TGC CCC TTC CA
*hGapdh*	GAT TTG GTC GTA TTG GGC	CTC GCT CCT GGA AGA TGG

### Determination of XO Activity and Uric Acid Levels

Xanthine oxidase activity and uric acid levels were measured by using specific and quantitative assays (Amplex Red Xanthine/Xanthine oxidase kit, and Amplex Red uric acid/uricase kit, respectively, both from Invitrogen) according to the manufacturer’s protocol.

### Xanthine Oxidoreductase Western-Blot

A goat polyclonal antibody against human XOR (diluted 1/500) and HRP conjugated polyclonal anti-goat antibody (1/2000, DAKO Cytomation, Glostrup, Denmark) were used as a primary antibody and secondary antibody, respectively. The cross-reacting signals were developed using the ECL Prime reagent (GE Healthcare UK Ltd.) and were visualized by a gel imaging system (ChemiDoc XRS+, Bio-Rad).

### Interleukin-6 Quantification

Cell supernatants were assayed using murine interleukin-6 (IL-6) ELISA kit (eBioscience) and by reading absorbance at 450 and 570 nm using the Spectramax M5e reader.

### Reactive Oxygen Species Level Measurement

Mitochondrial ROS level was measured with Red Mitochondrial Superoxide Indicator (MitoSOX, Life Technologies). Briefly, cells were stimulated for 2 h with 500 μg/ml hydroxyapatite (HA) crystals and then loaded 30 min with MitoSOX (at 5 μM final concentration), and fluorescence intensity measured (excitation 510 nm, emission 580 nm) using the Spectrax M5e reader.

### Glycosaminoglycans Detection

Glycosaminoglycans content was evaluated by Alcian blue staining at pH 2.5, allowing the identification of total glycosaminoglycans (without differentiation of carboxyl and sulfate groups). Briefly, chondrocytes or paraffin section of cartilage were fixed and stained with 1% Alcian blue 8GX in 3% acetic acid pH 2.5 (Sigma) for 30 min. Cells were washed in distilled water and images were captured. Quantification of blue staining was performed in Adobe Photoshop CC 2017 by number of positive pixels.

### LDH Measurement

Lactate dehydrogenase (LDH) in supernatant was measured using CytoTox-ONE^TM^ Homogeneous Membrane Integrity Assay (Promega) according to the manufacturer’s instructions. LDH release (%) was calculated by using the following formula. LDH release (%) = [(value in sample) − (background)]/[(value in Triton X-100-treated sample) − (background)] × 100.

### Statistical Analysis

Values represent means ± SD of triplicates. For each experiment, one representative experiment from a series of at least two independent experiments is shown. Data was analyzed with GraphPad Prism software. Variation between data sets was evaluated using the Student’s *t*-test or one-way or two-way ANOVA test, where appropriate. Differences were considered statistically significant at ^∗^*p* < 0.05, ^∗∗^*p* < 0.01, ^∗∗∗^*p* < 0.001, ^****^p < 0.0001.

## Results

### Pharmacological XOR Inhibition Attenuates Chondrocyte Mineralization and IL-6 Secretion *in vitro*

**FIGURE 1:**
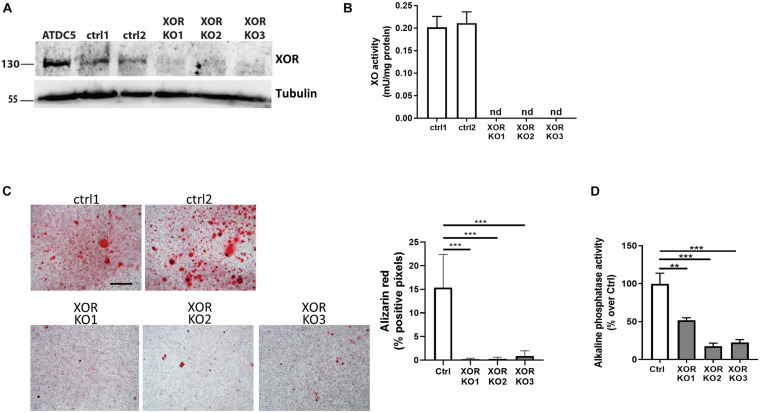
CRISPR/Cas9-mediated depletion of XOR leads to decreased chondrocyte mineralization. **(A)** Immunoblot analysis of XOR expression in cell extracts from ATDC5 cells, ATDC5 cell clones transduced with lentiviral control constructs (ctrl1, ctrl2), or lentiviral constructs with gRNA for XOR knock-out (XOR KO1, XOR KO2, XOR KO3). Equal amount of protein loading in the different samples was assessed by tubulin immunoblot. **(B)** XO activity in cell extracts from ATDC5 control (ctrl1 and ctrl2) and XOR knock-out (XOR KO1, XOR KO2, XOR KO3) clones. XO activity was determined by Amplex Red. **(C)** Alizarin red staining and quantification of Ctrl and XOR KO chondrocyte cell lines cultured with CPP for 24 h. Pictures are representative of one experiment of three independent experiments. Scale bar 400 μM. **(D)** Alkaline phospatase activity measurement in cell homogenates from Ctrl and XOR KO chondrocytes cultured with CPP for 6 hrs. ^∗∗^*p* < 0.01, ^∗∗∗^*p* < 0.001.

**FIGURE 2:**
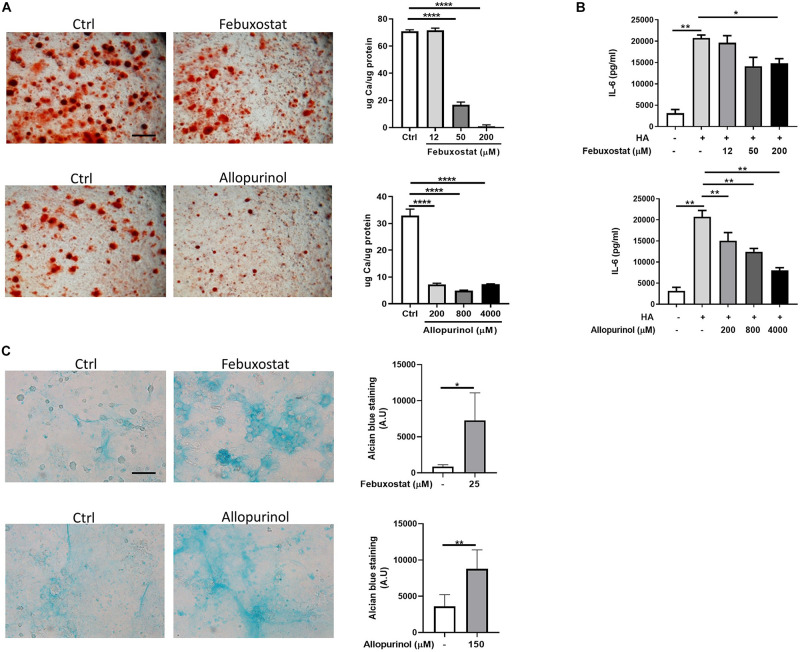
Xanthine oxidoreductase inhibitors regulate chondrocytes mineralization, IL-6 secretion, and extracellular matrix glycosaminoglycan content. **(A)** Representative Alizarin red staining of murine primary chondrocytes cultured with CPP and treated with 200 μM Febuxostat or 4 mM Allopurinol for 24 h. Pictures represent triplicates from one experiment of three independent experiments. Scale bar 400 μM. Graphs represent calcium content in the cell monolayer of chondrocytes cultured with CPP and treated with different doses of Febuxostat and Allopurinol for 24 h, expressed as μg Ca/μg protein. ^∗∗^*p* < 0.01, ^∗∗∗^*p* < 0.001, ^****^p < 0.0001. **(B)** IL-6 secretion in cell supernatant of chondrocytes stimulated for 24 h with HA crystals and different doses of Febuxostat or Allopurinol. ^∗^*p* < 0.05, ^∗∗^*p* < 0.01. **(C)** Representative Alcian blue staining of chondrocytes treated with 25 μM Febuxostat or 125 μM Allopurinol for 10 days. Pictures represent triplicates from one experiment of two independent experiments. Scale bar 100 μM. Graphs represent quantification of Alcian blue staining in the cell monolayer. ^∗^*p* < 0.05, ^∗∗^*p* < 0.01.

Primary murine chondrocytes expressed XOR as assessed by qRT-PCR (Δ*C*_*t*_ = 12) and by Western-blot (band at 130 kD, result not shown). Similarly, chondrogenic ATDC5 cells expressed XOR (Δ*C*_*t*_ = 14) by qRT-PCR and a 130 kD band by Western-blot (Figure 1A). To gain insights into the function of XOR in chondrocytes, two structurally unrelated XOR inhibitors, febuxostat and allopurinol were tested first in the chondrocyte mineralization assay. As expected from previous results (Nasi et al., 2020) primary chondrocyte calcification occurred after 24 h incubation with CPP. As demonstrated by Alizarin red staining (Figure 2A) and by quantification of calcium content in the cell monolayer (graph Figure 2A) calcification was strikingly attenuated by febuxostat. This inhibitory effect was recapitulated by allopurinol, the other XOR inhibitor. In parallel, we found that chondrocyte IL-6 secretion was also inhibited by both febuxostat and allopurinol in a dose-dependent manner (Figure 2B). These inhibitory effects were not due to effects on cell viability as no effect of these compounds on LDH levels were found (results not shown). The anti-mineralizing effect of XOR inhibitors were reproduced in the chondrogenic ATDC5 cell line (Supplementary Figure 1). These results support our previous finding that IL-6 sustains chondrocyte calcification (Nasi et al., 2016b), suggesting that XOR inhibitors impact mineralization by inhibiting IL-6 production. Increased mineralization has been associated with changes in extracellular matrix (ECM), and in particular in glycosaminoglycans (Hirschman and Dziewiatkowski, 1966; Chen et al., 1984). Indeed, we found by Alcian blue staining striking increase of glycosaminoglycans levels in primary chondrocytes upon febuxostat or allopurinol treatment for 10 days (Figure 2C).

### Xanthine Oxidoreductase Deficiency Inhibited Chondrocyte Mineralization *in vitro*

As primary murine chondrocytes, also chondrogenic ATDC5 cells expressed XOR (Δ*C*_*t*_ = 14) by qRT-PCR and a 130 kD band by Western-blot (Figure 1A). To further test the role of XOR in chondrocytes, we generated XOR knock-out cells using a lentiviral CRISPR/cas9 system. ATDC5 cells were transduced with lentiviral constructs with control guided RNAs or with guided RNAs targeting XOR. The gRNAs designed to knock-out XOR expression were efficient as indicated by disappearance of both XOR protein expression by Western-blot (Figure 1A) and XO activity (Figure 1B) in three independent XOR KO clones. Control and XOR KO clones had the same proliferation capacities (Supplementary Figure 3A). These clones were further studied for their calcification potential. XOR KO clones had a reduced capacity to mineralize compared to controls (Figure 1C). In addition to a decreased mineralization capacity, XOR KO clones also showed decreased Alp activity (Figure 1D).

Since the behavior of the three XOR KO clones was reproducible concerning mineralization and Alp activity, we next focused on one of them: XOR KO1 cell line. As expected, XOR KO1 cells had significantly reduced pro-mineralizing IL-6 (Supplementary Figure 3B) and uric acid levels compared to control cells (Supplementary Figure 3C). We also found that upon HA crystal stimulation, a known stimulus of ROS production in macrophages (Ives et al., 2015) and chondrocytes, ROS levels were significantly induced in control Ctlr1 cells but not in XOR KO1 chondrocytes (Supplementary Figure 3D). This was not due to decreased viability of XOR KO1 cells upon HA stimulation (see MTT assay and Supplementary Figure 3A).

We next investigated whether XOR deficiency could affect ECM composition and degradation. We found in control and XOR KO1 chondrocytes similar levels of collagen II, and of the hypertrophic chondrocyte marker collagen X (as assessed by immunohistochemistry, Supplementary Figures 3E,F). Finally, MMP13, a critical protease involved in ECM degradation was also unchanged compared to control chondrocytes (Supplementary Figure 3G).

### The XO and XDH Forms of XOR Differ in Mineralization and IL-6 Secretion

**FIGURE 3:**
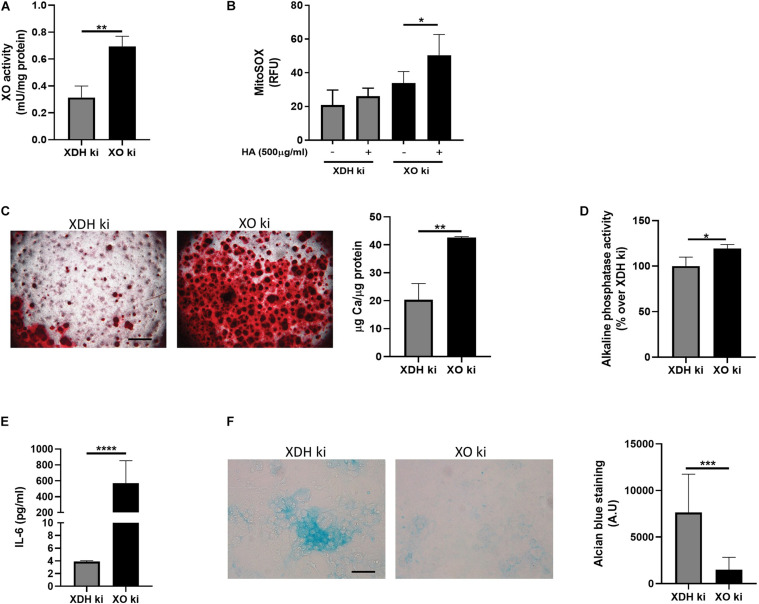
Xanthine oxidase form of XOR is preferentially involved in chondrocyte mineralization, ROS production and IL-6 secretion. **(A)** XO activity measurement by AmplexRed Kit in cell homogenates from XDH ki and XO ki chondrocytes. ^∗∗^*p* < 0.01. **(B)** Mitochondrial ROS production in XDH ki and XO ki chondrocytes stimulated or not with 500 μg/ml HA crystals for 2 h. ^∗^*p* < 0.05. **(C)** Representative Alizarin red staining of XDH ki and XO ki chondrocytes cultured with CPP for 24 h. Pictures from one experiment of three independent experiments are shown. Scale bar 400 μM. Graphs represent calcium content in the cell monolayer of XDH ki and XO ki chondrocytes cultured with CPP for 24 h, expressed as μg Ca/μg protein. ^∗∗^*p* < 0.01. **(D)** Alkaline phosphatase activity measurement in cell homogenates from XDH ki and XO ki chondrocytes cultured with CPP for 6 h. ^∗^*p* < 0.05. **(E)** IL-6 secretion in cell supernatant of chondrocytes from point (c). ^****^p < 0.0001. **(F)** Representative Alcian blue staining of XDH ki and XO ki chondrocytes cultured for 10 days. Pictures represent triplicates from one experiment of two independent experiments. Scale bars 100 μM. Graphs represent quantification of Alcian blue staining in the cell monolayer. ^∗∗∗^*p* < 0.001.

We and others found that H_2_O_2_ stimulation led to significantly increased calcification, while the ROS scavenger NAC reverted the latter effect (Morita et al., 2007; Nasi et al., 2020). We therefore hypothesized that the pro-mineralizing effects of XOR could be mediated by ROS generation via the XO form of XOR. We took advantage of the previously generated *Xdh* gene-modified mice, expressing either knocked-in XDH stable (C995R) mutant protein (XDH ki), or knocked-in XO-locked (W338A/F339L) mutant protein (XO ki). We isolated from these mice primary chondrocytes expressing mainly the XDH form (XDH ki) or exclusively the XO form (XO ki). As expected, primary XO ki chondrocytes possessed significantly more XO activity compared to XDH ki chondrocytes at the basal state (Figure 3A). Upon stimulation with HA crystals, they produced significantly more ROS than XDH ki chondrocytes (Figure 3B). Taken together, these results confirmed that XO ki chondrocytes overproduced ROS.

Considering that high ROS levels are reported to inhibit proliferation and modulate the initiation of hyperthrophic changes in chondrocytes (Morita et al., 2007), we assessed the contribution of the ROS-generating XO form to the calcification potential of primary chondrocytes. We found a predominant mineralizing phenotype in XO ki chondrocytes compared to XDH ki chondrocytes, as evidenced by enhanced alizarin red staining (Pictures, Figure 3C) and increased calcium content (Histogram, Figure 3C). Increased mineralization in XO ki chondrocytes was associated with enhanced Alp activity (Figure 3D) and IL-6 secretion (Figure 3E). Accordingly, we found decreased proliferation of XO ki chondrocytes compared to XDH ki chondrocytes (Supplementary Figure 2B). Moreover, glycosaminoglycans content as evidenced by Alcian blue staining was decreased in XO ki chondrocytes (Figure 3F), thus reinforcing the negative correlation between proteoglycan content and mineralization. However, other markers of chondrocyte differentiation in XO ki chondrocytes such as collagen II, collagen X or MMP13 were similarly expressed in XO ki and XDH ki chondrocytes (Supplementary Figure 4).

Altogether, our study suggests that XO, via ROS generation, exacerbates chondrocyte mineralization. In addition, XO ki chondrocytes have the capacity to generate more ROS upon HA-crystal stimulation.

### Xanthine Oxidoreductase Expression Is Increased in Human OA Cartilage

**FIGURE 4:**
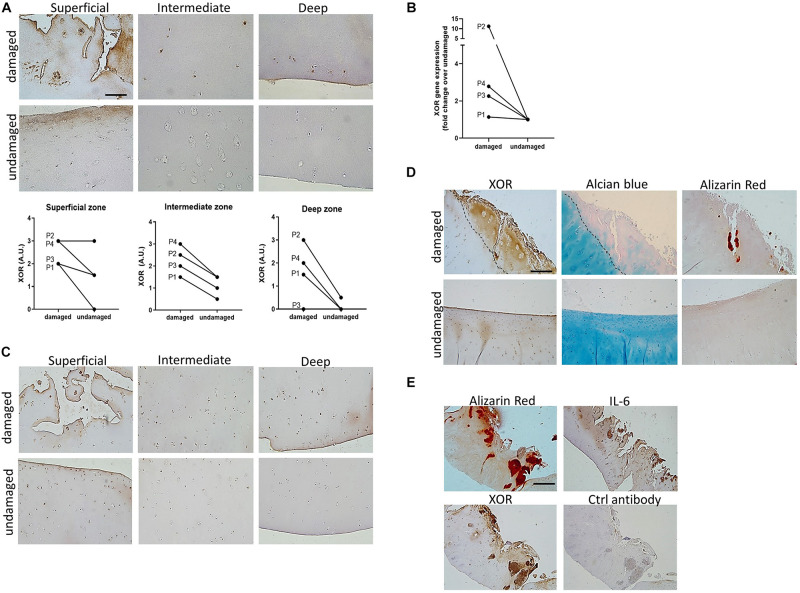
Cartilage XOR expression correlates with cartilage damage and calcification in OA. **(A)** XOR immunohistochemical staining in superficial, intermediate and deep zone of macroscopically damaged and undamaged cartilage from end-stage osteoarthritis patients. One representative picture from one out of four patients is shown. Scale bar 200 μm. Graph represent quantification of XOR staining in damaged and undamaged cartilage from four OA patients (named P1, P2, P3, and P4). **(B)** qRT-PCR analysis of XOR gene expression in damaged and undamaged cartilage from patients in point (a). **(C)** IL-6 immunohistochemical staining in superficial, intermediate and deep zone of macroscopically damaged and undamaged cartilage from end-stage osteoarthritis patients. One representative picture from one out of four patients is shown. Scale bar 200 μm. Sections are consecutive to those in panel **(A)**. **(D)** XOR immunohistochemical staining in cartilage and consecutive sections stained with Alcian blue staining for glycosaminoglycans and Alizarin Red staining for calcium-containing crystals. For each staining, one representative picture from one out of four patients is shown. Scale bar 400 μm. **(E)** Alizarin Red staining for calcium-containing crystals in cartilage and consecutive sections stained with IL-6 and XOR immunohistochemistry or a control (Ctrl) antibody. For each staining, one representative picture from one out of four patients is shown. Scale bar 800 μm.

We dissected macroscopically undamaged and damaged pieces of articular cartilage from the femoral condyles of 4 OA patients undergoing total knee replacement surgery, and analyzed them for XOR expression. Immunohistochemistry of undamaged cartilage revealed XOR expression by chondrocytes in the superficial and intermediate cartilage layers but not in deep layers. In damaged cartilage, XOR expression was increased in all cartilage areas analyzed (Figure 4A). We used an anti XOR antibody directed against an epitope corresponding to amino acids 251–360 mapping near the N-terminus of XOR, thus recognizing both XO and XDH forms. The staining with this antibody was specific as demonstrated by controls with non-immune rabbit IgG 5 ug/ml (in place of anti XO IgG 5 ug/ml, see Figure 4E) or absence of primary antibody (results not shown) in which very little if any staining was noted. In agreement with these immunohistological results, XOR mRNA levels were increased in damaged versus undamaged osteoarthritic cartilage (Figure 4B). We next analyzed IL-6 expression on consecutive slides and found expression pattern similar to those of XOR (Figure 4C, i.e., in undamaged cartilage IL-6 expression by chondrocytes in the superficial and intermediate cartilage layers but not in deep layers; in damaged cartilage, increased IL-6 expression in all cartilage layers). The glycosaminoglycans content of the damaged cartilage was reduced compared to undamaged tissue as evidenced by Alcian blue staining (Figure 4D). Finally, in all the damaged cartilage samples examined, we observed the presence of alizarin red positive calcium-containing crystals. Interestingly, both XOR expression and IL-6 expression was colocalized with some of the crystal deposits (Figures 4D,E).

Altogether, these results indicate that chondrocyte XOR expression increases with the severity of cartilage damage in human OA, and is correlated with cartilage calcification.

## Discussion

A large body of evidence supports the idea that calcium-containing crystals are active players in the initiation and progression of OA (Conway and McCarthy, 2018; McCarthy and Dunne, 2018). Clinically, calcification is commonly seen in the context of aging, joint trauma, conditions that are associated with OA. The mechanisms that lead to this pathological calcification are still not well understood.

Local inflammation and chondrocyte ROS levels influence chondrocyte mineralization (Morita et al., 2007). Here, we have demonstrated for the first time that XOR is expressed by chondrocytes in culture and *in vivo*, and plays a role in cartilage calcification. XOR is a major enzyme in the purine degradation pathway and the XO form is in addition a source of cellular ROS. XO is formed under conditions of cellular stress or inflammation (Parks et al., 1988; Battelli et al., 2018). Our results showed that when XO activity is reduced, either by the administration of XO inhibitors or by deletion of the *xdh* gene, there is a reduction of chondrocyte mineralization as well as IL6 secretion. The availability of genetically modified mice that expressed selectively only one form of XOR allowed us to study the role of the XO vs. the XDH form in calcification. The XO form exhibited increased calcification, as well as increased IL6 production with increased cellular ROS levels, particularly when chondrocytes were cultured in the presence of calcium crystals (HA crystals). Our data further highlight the cross-talk between calcification and ROS production in chondrocytes. We have shown before that H_2_O_2_ induced calcification in chondrocytes and that the ROS scavenger NAC could revert this effect (Nasi et al., 2020). On the other hand, HA crystals found in chondrocyte calcification induced both cytosolic and mitochondrial ROS (Nasi et al., 2016a). Our results generated with XOR KO chondrocytes further confirm the reciprocal link between calcification and ROS production, as we found that XOR deficient chondrocytes had a reduced capacity to induce ROS and to mineralize. In addition, in the mutated chondrocytes expressing the XDH form, unable to produce ROS, we found a decrease capacity to generate ROS and reduced calcification compared to chondrocytes expressing the ROS-producing the XO form. XOR-derived ROS (primarily H_2_O_2_ and superoxide) play a role in chondrocyte mineralization. However, there are other potential sources of ROS in chondrocytes (e.g., mitochondrial respiratory enzymes and NADPH oxidase) that could contribute to increased oxidative stress and increased mineralization. Conversely, anti-oxidant enzymes such as superoxide dismutase could decrease oxidative stress and mineralization. For instance tempol, a superoxide dismutase mimetic agent ameliorated vascular smooth muscle cell calcification and arterial medial calcification in uremic rats, together with reduction in aortic and systemic oxidative stress levels (Yamada et al., 2012). Further studies are required to determine the collective roles of the enzymes involved in oxidative stress and chondrocyte mineralization.

In addition to ROS, another pro-mineralizing player is IL-6. IL-6 is indeed a strong up-modulator of Alp, a crucial enzyme involved in the mineralization process (Bastidas-Coral et al., 2016). In addition, IL-6 can also impact other pro-mineralizing molecules such as Annexin5, Ank and Pit1 and on inflammation (Nasi et al., 2016b; Ehirchiou et al., 2020). Finally, Alp, IL-6 and mineralization have all been associated to OA development and progression (Nasi et al., 2016b; Park et al., 2020). Here we demonstrated that both IL-6 secretion and Alp were significantly up-regulated in XO ki versus XDH ki chondrocytes, and that Febuxostat and Allopurinol inhibited HA-induced IL-6 secretion. Interruption of this mineralization triggers by XOR inhibitors could therefore be beneficial in slowing the progression of OA. In support of this, Aibibula et al. (2016) reported that treatment with febuxostat reduced major OA features in a murine model of diet-induced OA.

Xanthine oxidase activity also modulated proteoglycan turnover. Previous studies reported that addition of XO to articular cartilage in culture resulted in decreased proteoglycan synthesis (Bates et al., 1984). In addition it was found that H_2_O_2_ was the active ROS involved in this phenomenon (Bates et al., 1984; Schalkwijk et al., 1985). In our study, XO ki chondrocytes had significantly lower levels of glycosaminoglycans. Accordingly, we found that XO inhibitors significantly increased proteoglycan content of chondrocytes. However, we did not find significant changes in the expression of collagen II and collagen X matrix components in XDH ki versus XO ki chondrocytes.

Uric acid plays a complex role in cartilage pathophysiology. It was reported that physiological concentrations of uric acid had both anti-inflammatory and chondroprotective effects *in vitro* and *in vivo* (Lai et al., 2017). On the other hand several independent observations indicated that hyperuricemia was associated with more severe OA and predicted OA progression (Denoble et al., 2011; Krasnokutsky et al., 2017; Neogi et al., 2019). In these studies, higher uric acid levels could partly reflect increased XOR activity in cartilage, leading ultimately to deleterious effects on cartilage integrity. In our study, we observed that damaged OA cartilage expressed higher levels of XOR than undamaged cartilage, which co-localized with calcium crystal deposits in some samples. This strongly suggests that local XOR expression and activity participate in cartilage calcification and damage in OA. It is likely that in the context of high oxidative stress such as in OA cartilage, XO form would be preferentially expressed. New antibodies discriminating XO versus XDH forms, will be useful tools to identify if XO is crucially involved in cartilage calcification and degradation in OA. Taken together, our findings suggest that XOR inhibitors may be of therapeutic benefit in OA.

## Data Availability Statement

The raw data supporting the conclusions of this article will be made available by the authors, without undue reservation.

## Ethics Statement

The studies involving human participants were reviewed and approved by the Centre Hospitalier Universitaire Vaudois ethical committee, Lausanne, Switzerland. The patients/participants provided their written informed consent to participate in this study. The animal study was reviewed and approved by the Service de la consommation et des affaires vétérinaires du Canton de Vaud, Switzerland.

## Author Contributions

SN designed, performed, and evaluated most experiments. MC generated the XOR knockout chondrocyte cell lines and performed part of the experiments. VC performed the histological/immunohistological analysis. DE set up and performed the FACS analysis. TK, TN, and KO generated the mutated XOR mice. IB performed the human cartilage extraction, qPCR analysis, and staining quantifications. AS and NB designed the project and evaluated results. All authors participated to the writing of the manuscript.

## Conflict of Interest

The authors declare that the research was conducted in the absence of any commercial or financial relationships that could be construed as a potential conflict of interest.

## Abbreviations

Alp: alkaline phosphatase
BCP: basic calcium phosphate
CPPD: Calcium pyrophosphate dihydrate
CPP: secondary calciprotein particles
ECM: extracellular matrix
HA: hydroxyapatite
IL-6: interleukin-6
Mmp-13: metalloprotease-13
MNX: meniscectomy
OA: osteoarthritis
ROS: reactive oxygen species
XOR: xanthine oxidoreductase
XO: xanthine oxidase
XDH: xanthine dehydrogenase.
