# Supplementary figures and images for: Xanthine Oxidoreductase Is Involved in Chondrocyte Mineralization and Expressed in Osteoarthritic Damaged Cartilage
Source: Front Cell Dev Biol. 2021 Feb 9;9:612440. doi: 10.3389/fcell.2021.612440 (PMC7900416; doi:10.3389/fcell.2021.612440)

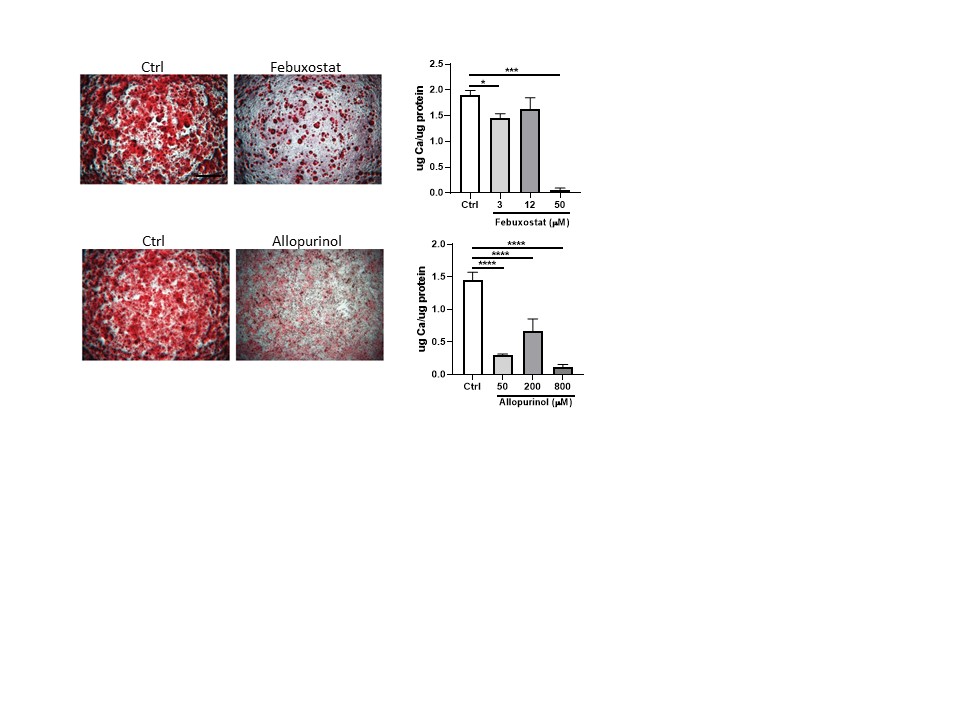

Supplement: Supplementary file 1 [file Image_1.jpg]

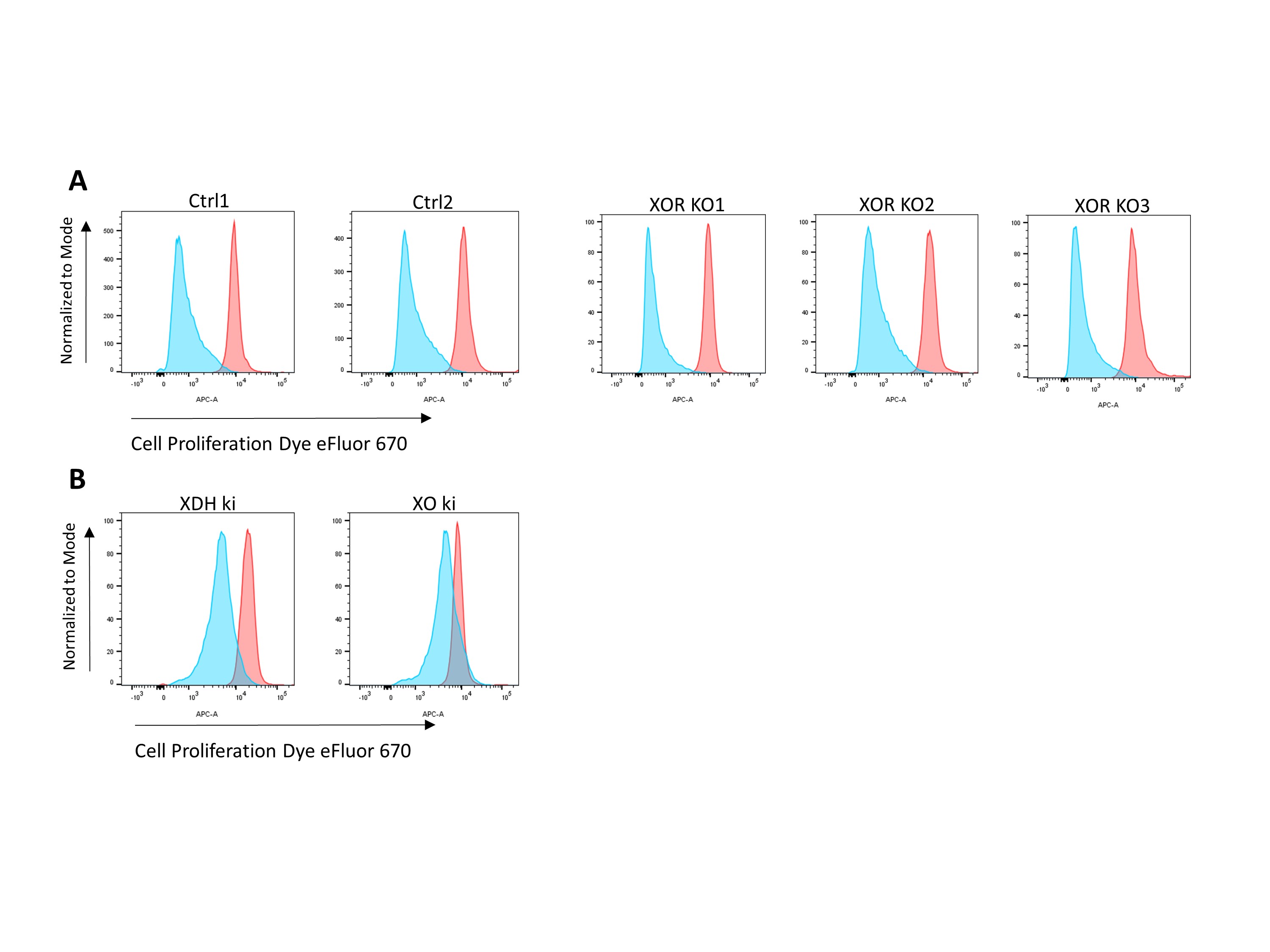

Supplement: Supplementary file 2 [file Image_2.JPEG]

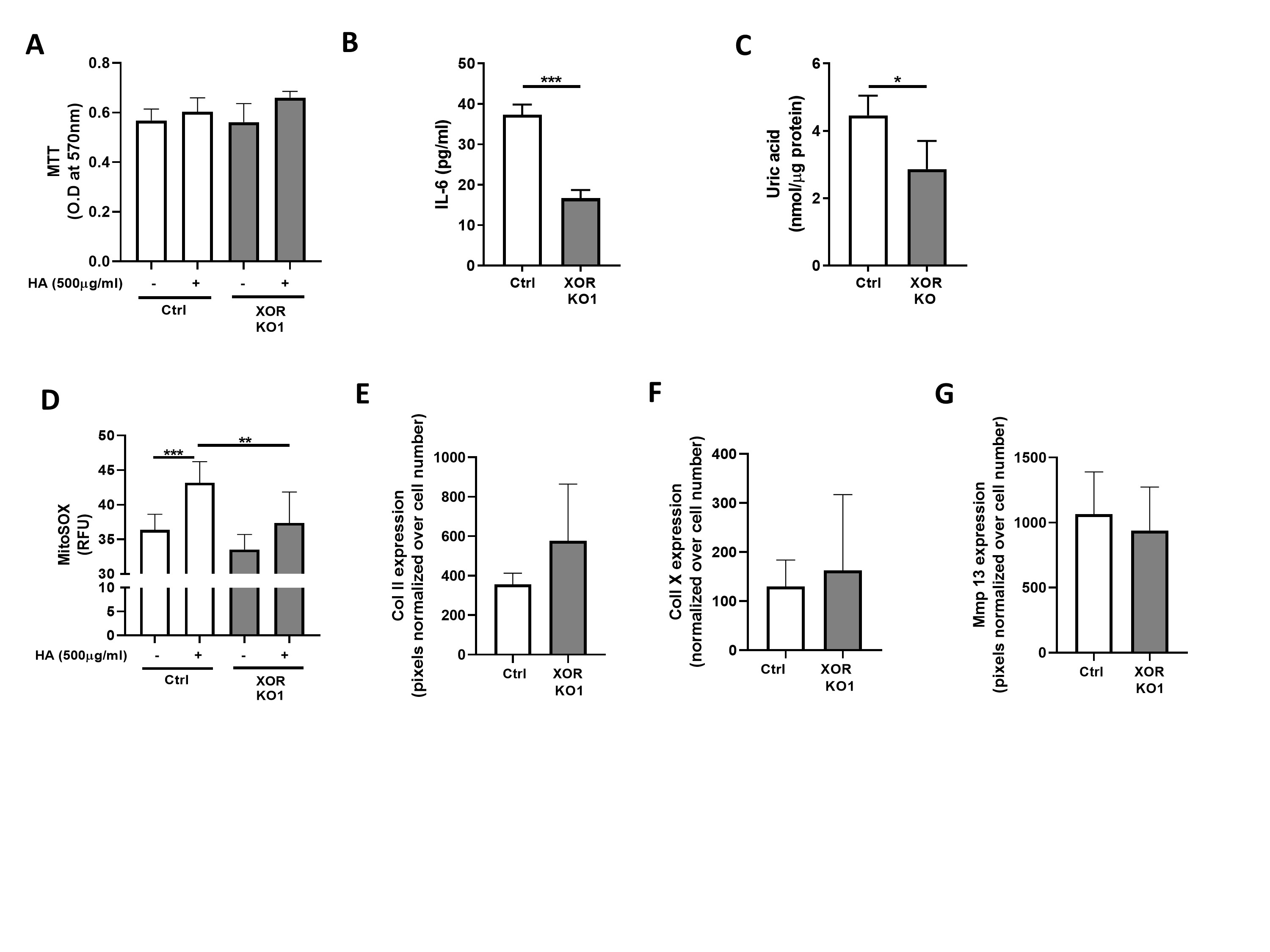

Supplement: Supplementary file 3 [file Image_3.JPEG]

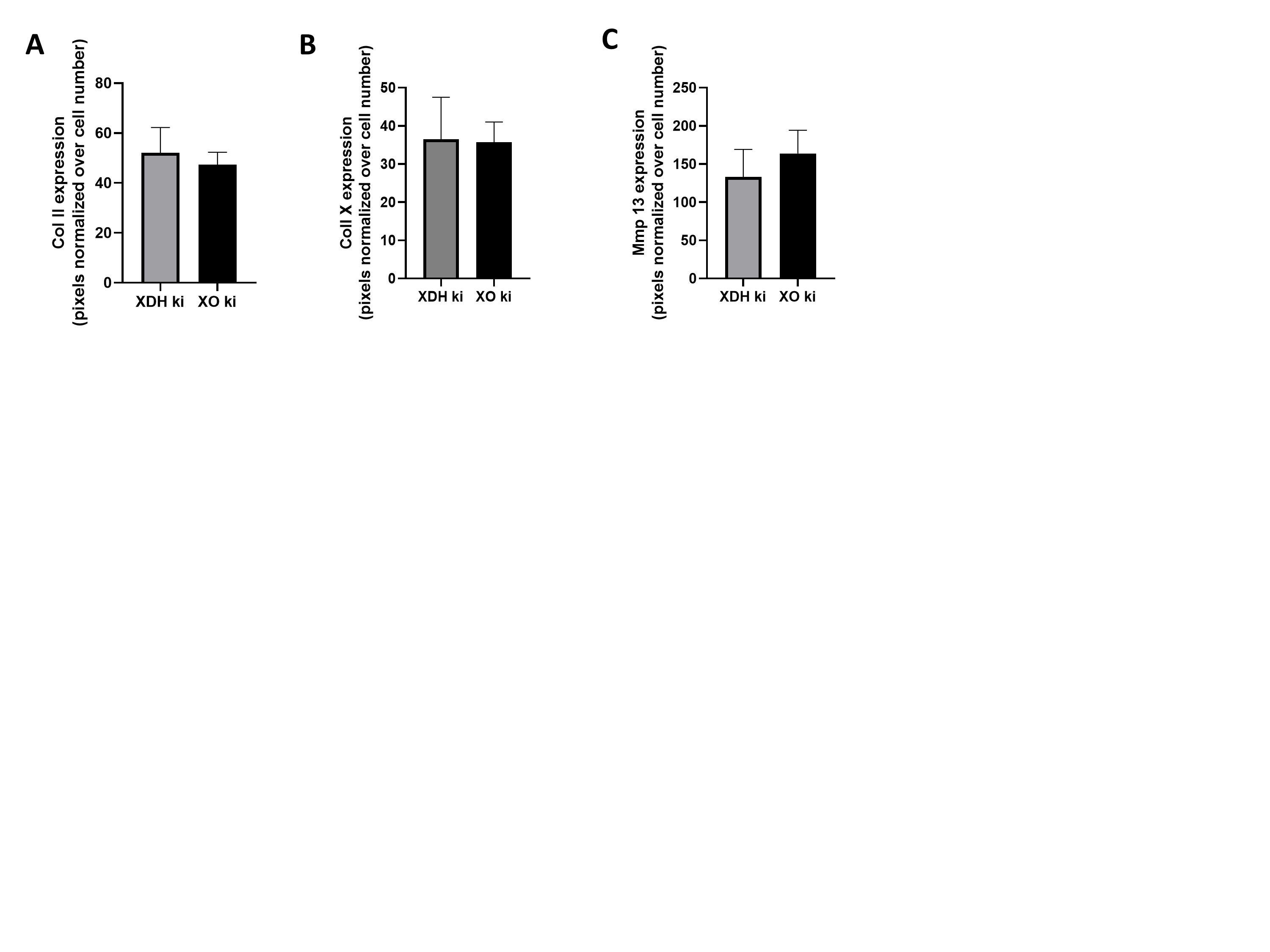

Supplement: Supplementary file 4 [file Image_4.JPEG]
